# Cancer surveillance in biliary atresia patients with native liver survival: Standardizing monitoring and follow-up

**DOI:** 10.1007/s00383-025-06066-y

**Published:** 2025-06-12

**Authors:** Omid Madadi-Sanjani, Lutz Fischer, Marie Uecker, Christian Tomuschat, Uta Herden, Martina Sterneck, Bianca Hegen

**Affiliations:** 1 Department of Visceral Transplantation, University Medical Center Hamburg-Eppendorf, Martinistreet 52, 20251 Hamburg, Germany; 2 Department of Pediatric Surgery, Hannover Medical School, Hannover, Germany; 3 Department of Pediatric Surgery, University Medical Center Hamburg-Eppendorf, Hamburg, Germany; 4 I. Department of Internal Medicine, University Medical Center Hamburg-Eppendorf, Hamburg, Germany; 5 Department of Pediatric Gastroenterology and Hepatology, University Childrens Hospital, University Medical Center Hamburg-Eppendorf, Hamburg, Germany

**Keywords:** Biliary atresia, Native liver survival, Hepatocellular carcinoma, Hepatoblastoma, Cholangiocarcinoma

## Abstract

Biliary atresia (BA) is a rare cholangiopathy in neonates, leading to end-stage liver failure in the first years of life, when left untreated. Kasai procedure aims to restore biliary drainage to achieve native liver survival. While liver transplantation is the necessary treatment in children with failure of the Kasai procedure, the number of native liver survivors in the long-term remains around 20–30%. Reports on malignancies in native livers of children, adolescents and adults with BA are increasing, including cases of hepatocellular carcinoma, cholangiocarcinoma, hepatoblastoma and small intestinal adenocarcinoma. In this review we summarize the reports, with focus on tumor markers, imaging technologies and reported outcomes. Furthermore, we discuss recent advances in surveillance strategies in adults with chronic liver impairment.

## Introduction

Biliary atresia (BA) is a rare fibro-obliterative cholangiopathy of unknown etiology [1]. Untreated, the disease progresses to extra- and intrahepatic bile duct destruction resulting in cholestatic end-stage liver disease [2]. Early diagnosis of BA is crucial for the prognosis. The primary intervention is the Kasai procedure (KPE) where biliary drainage in the jaundiced neonates is surgically restored with the aim of prolonging native liver survival [3]. Whilst 50–60% of children will achieve a temporary clearance of jaundice following KPE, 70–80% will undergo liver transplantation before reaching adulthood, making BA the most common indication for pediatric liver transplantation [4]. In children with clearance of jaundice and long-term native liver survival the liver fibrosis still progresses, affecting patients’ health-related quality of life and requiring regular multidisciplinary follow-ups for management of cirrhosis, portal hypertension and its sequelae [5].

Chronic liver disease and inflammation are well-known risk factors for malignant transformation, given the association of chronic hepatitis B and C, alcohol-related liver disease and non-alcoholic fatty liver disease with hepatocellular carcinoma (HCC) or primary sclerosing cholangitis, bile duct stones or cysts with cholangiocarcinoma (CCA) in adults [6–8]. With the improved overall survival in BA affected children, the monitoring for malignancies in survivors with native liver following KPE has gained importance [9, 10]. Reports on liver and bile duct malignancies in BA cohorts are becoming more frequent, with an estimated incidence of HCC in these patients of 1–2% [11]. However, appropriate monitoring and screening measures are lacking, with scarce data on laboratory markers or radiological findings.

We present an overview of the literature on malignancies in native liver survivors with biliary atresia and discuss current diagnostic concepts and instruments that might be suitable for pediatric patient groups.

## Methods

A literature search was performed using PubMed and EMBASE using the keywords biliary atresia AND malignancy, carcinoma, cancer, hepatocellular carcinoma, cholangiocarcinoma, hepatoblastoma and sarcoma. The articles and their reference lists were screened for additional publications. The literature search via the databases was conducted in January and March 2024.

**Fig. 1:**
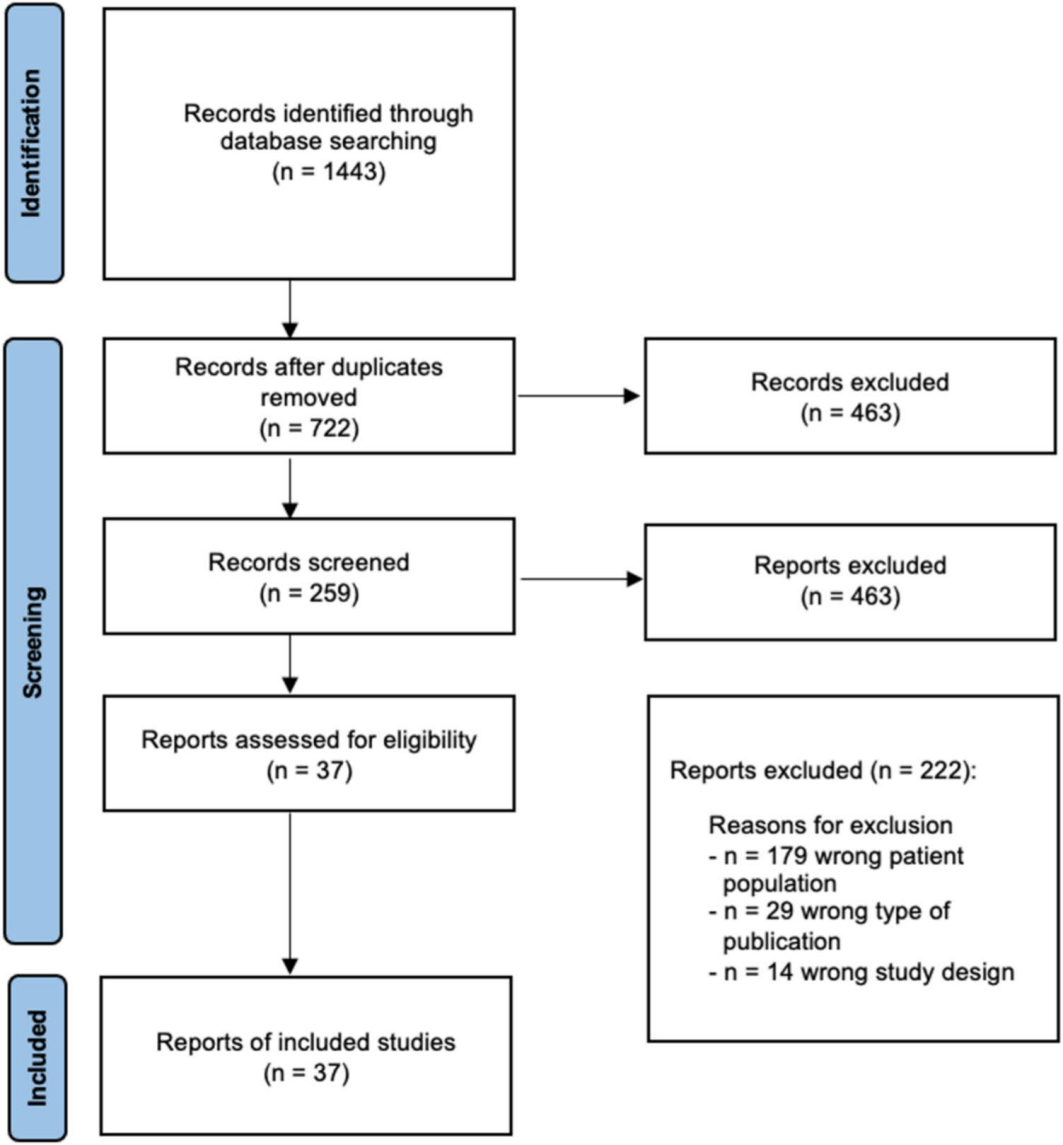
PRISMA flowchart. *PRISMA* Preferred reported items for systematic reviews and meta-analysis

The review was performed according to the Preferred Reported Items for Systematic Reviews and Meta-Analysis (PRISMA) guidelines. Two reviewers (OMS, BH) independently screened the abstracts of the studies retrieved using the search strategies (Fig. 1).

### Eligibility criteria

All case reports and original studies reporting on the diagnosis of malignancies in BA survivors with native liver were considered eligible, when an English abstract was available. Reports on post-transplant malignancies were excluded.

### Data extraction

The following data were extracted by the reviewers: Data regarding the number of cases reported in the manuscript (n =), initial surgical procedure (Kasai-procedure [KPE], primary liver transplantation [pTLx], Choledocho-jejunostomy [CD], hepatico-jejunostomy [HJ], none), carcinoma type (hepatocellular carcinoma [HCC], cholangiocarcinoma [CCA], hepatoblastoma [HB], adenocarcinoma [ADC], papillary thyroid cancer [PTC]), the time of diagnosis [before transplant [pre-LTx], incidental histological finding of transplant hepatectomy specimen [incidental at LTx], or independent from surgery [no surgery, post mortem autopsy]), diagnostics (computed tomography [CT], magnet resonance imaging [MRI], ultrasound [US], contrast-enhanced ultrasound [CEUS], positron-emission tomography-computed tomography [PET/CT], Endoscopy with cytology or mapping biopsy, endoscopic ultrasound [EUS], alpha-fetoprotein [AFP], carcinoembryonal antigen [CEA], carbohydrate antigen 19–9 [CA19-9], exploratory laparotomy), defined as positive if carcinoma signs were present, the age at diagnosis (years [yr], months [mo]), liver function (liver failure with progressive jaundice [LF], native liver survivors [SNL], jaundice-free native liver survivors [JF-SNL]), the outcome depending on post-treatment tumor recurrence and information.

## Results

A total of 37 publications, with 57 cases of carcinomas (HCC: n = 42; CCA n = 8; Hepatoblastoma n = 5; others n = 2) were identified. All manuscripts were retrospective analyses, mostly referring to case series and case reports (Oxford Evidence-Based Medicine Level 4).

### Hepatocellular carcinoma (HCC)

#### Patient information

Information on 42 HCC diagnosis (73.7%; 42/57) in BA patients with native liver were extracted from 26 reports (Table 1). Twenty-seven patients (64.3%) underwent KPE as first-line treatment during early childhood, three (7.1%) received pLTx, in one (2.4%) a choledocho-duodenostomy for correctable biliary atresia (Ohi type I) was performed, and in the additional 11 (26.2%) either no procedure was performed (historic reports before 1971) or no information were available.

**Table 1:** Summary of 42 hepatocellular carcinoma (HCC) cases in biliary atresia patients from 26 reports

Author	N =	Procedure	Condition at KPE	Post-KPE complications	Timing of diagnosis	Histological type	Imaging	Tumor markers	Age	Liver tests	Outcome
Kawaguchi et al. 2023 [49]	1	KPE	Age: 67d	n.a	Pre-LTx	n.a	n.a	n.a	34 yr	n.a	Deceased
Vij et al. 2023 [46]	1	KPE	Age: 77d	n.a	Pre-LTx	*Syncytial giant cell variant*	CT: pos	AFP: pos	7mo	LF	n.r.: 8mo post-LTx
Calinescu et al. 2022[50]	2	KPE	n.a	n.a	Incidental at LTx	*Well-differentiated*	US: neg CT: neg	n.a	1 yr (I) 15 yr(II)	n.a	n.r.*
Özcay et al. 2022 [51]	1	n.a	n.a	n.a	n.a	n.a	n.a	AFP: neg	7 yr	n.a	n.r.*
Parolini et al. 2019 [52]	1	KPE	n.a	n.a	Incidental at LTx	n.a	n.a	n.a	7 yr	n.a	n.a
Vinayak et al. 2017 [53]	1	KPE	n.a	n.a	Incidental at LTx	n.a	n.a	n.a	7 yr	n.a	n.a
Squires et al. 2017 [54]	1	KPE	Age 4w	Portal hypertension	Pre-LTx	n.a	US: pos CEUS: pos MRI: pos	AFP: neg	13mo	JF-SNL	n.a
Hirzel et al. 2015 [55]	2	KPE	Age: n.a. (= 1) 40d (n = 1)	n.a	Pre-LTx	*Well-differentiated* (pT2, pNx) (n = 2)	US: pos MRI: pos CT: pos	n.a	4 yr (I) 25 yr (II)	LF (n = 1)	n.r.: 1 yr (I) 2mo (II)
Yoon et al. 2014 [56]	2	KPE	n.a	n.a	Pre-LTx	*Well-differentiated*	CT: pos MRI: pos	AFP: neg	7mo (I) 16 yr (II)	n.a	n.r.: 20 m post LTx (I) 15 m post RT (II)
Zen et al. 2014 [57]	3	KPE (n = 2) pLTx (n = 1)	n.a	n.a	Pre-Tx (n = 1) Incidental at LTx (n = 2)	*Well- to moderately differentiated*	CT: pos (n = 1)	AFP: pos (n = 2)	1 yr (I) 2 yr (II) 2 yr (III)	n.a	n.r.: 4 yr 7 yr 5 yr
Aggarwal et al. 2012 [58]	1	KPE	Age: 3 m	n.a	Pre-LTx	n.a	CT: pos	n.a	23 yr	LF	Listed for transplant
Kim et al. 2012 [47]	1	KPE	Age: 3 m	n.a	Incidental at LTx	*Well-differentiated*	n.a	n.a	10mo	LF	n.r.: 4mo post-LTx
Hadzic et al. 2011 [11]	5	KPE (n = 4) pLTx (n = 1)	Age: 66d 71d 68d 49d	Portal hypertension (n = 2)	Pre-Tx (n = 3) Incidental at LTx (n = 2)	*Well-differentiated* (n = 2) *Moderately-differentiated* (n = 3)	MRI: neg (n = 1) CT: pos (n = 2) US: neg (n = 1)	AFP: neg (n = 2) AFP: pos (n = 1)	40mo (I) 26mo (II) 24mo (III) 14 yr (IV) 12mo (V)	LF (n = 3) SNL (n = 2)	n.r.: 2.5 yr (I) 4.1 yr (II) 2.5 yr (III) 5.7 yr (IV) 2 yr (V)
Romano et al. 2011 [48]	3	n.a	n.a	n.a	Pre-LTx (n = 2) Incidental at LTx (n = 1)	*Well-differentiated* (n = 1) *Moderately-differentiated* (n = 1) *Poorly-differentiated* (n = 1)	n.a	AFP: pos (n = 2) AFP: neg (n = 1)	0.7 yr (I) 8 yr (II) 3.6 yr (III)	JF-SNL (n = 1) LF (n = 2)	Deceased (n = 1) n.r.: (n = 2) 1.6 yr (I) 4.0 yr (II)
Iida et al. 2009 [59]	1	pLTx	n.a	n.a	Incidental at LTx	*Moderately-differentiated* (pT2, N0, M0, stage2)	n.a	AFP: pos	10mo	LF	n.r.: 4mo post-LTx
Hol et al. 2008 [60]	1	KPE	n.a	n.a	Pre-Tx	*Well-differentiated* (pT1, N0, M0)	MRI: pos	AFP: neg CEA: neg	19 yr	LF	n.r.: 3 yr post-LTx
Brunati et al. 2007 [61]	1	KPE	Age: 8w	Portal hypertension	Pre-LTx	n.a	US: pos MRI: neg	AFP: pos	8mo	LF	n.r.: 1 yr post-LTx
Tatekawa et al. 2001[12]	3	KPE	Age: 66d 70d 47d	Portal hypertension (n = 2)	Pre-LTx	*Moderately-differentiated* (T4, N0, M0, stage 4a) (n = 1) *Well-differentiated* (T2, N0, M0, stage 2) (n = 1) *Poorly-differentiated* (T4, N0, M0, stage 4a)	CT: pos	AFP: pos (n = 2) AFP: neg (n = 1)	8 yr 10 yr 4 yr	LF (n = 2)	n.r.: 5 yr 2 yr n.a
Sato et al. 2000 [62]	1	KPE	n.a	n.a	Pre-LTx	n.a	US: pos	n.a	3 yr	n.a	n.a
Superina et al. 1996 [63]	1	KPE	n.a	n.a	Pre-LTx	n.a	US: pos	n.a	12 yr	JF-SNL	n.r.: 3 yr post-LTx
Kohno et al. 1995 [64]	1	KPE	Age: 2 m	Portal hypertension	Pre-LTx	*Well-differentiated*	US: pos	AFP: pos	4 yr	LF	Deceased
Esquivel et al. 1994 [65]	2	n.a	n.a	n.a	Pre-LTx (n = 1) Incidental at LTx (n = 1)	n.a	n.a	AFP: pos (n = 1)	15mo (I) 11 yr (II)	n.a	n.r.: 34mo (I) 44mo (II)
Van Wyk et al. 1972 [66]	2	CD (n = 1) None (n = 1)	Age: n.a. (n = 1) 5w (n = 1)	Portal hypertension (n = 2)	Pre-Tx (n = 1) Incidental at LTx (n = 1)	*Well-differentiated* (n = 1)	n.a	AFP: pos	12 yr (I) 4 yr (II)	LF	Deceased (LTx rejection) (n = 1) n.r. (n = 1): 18mo post-LTx
Starzl et al. 1971 [67]	2	n.a	n.a	n.a	Pre-LTx (n = 1) Incidental at LTx (n = 1)	n.a	n.a	AFP: pos	10 yr (I) 4 yr (II)	n.a	Deceased (n = 1) n.r. (n = 1): 9mo post-LTx
Deoras et al. 1968 [68]	1	n.a	n.a	n.a	No surgery Post mortem autopsy	n.a	n.a	n.a	6 yr	n.a	Deceased
Okuyama 1965 [69]	1	None	n.a	n.a	No surgery Post mortem autopsy	n.a	n.a	n.a	3 yr	LF	Deceased

*n.a*. not available, *n.r*. no recurrence, *LTx* liver transplantation, *JF-SNL* jaundice-free survival with native liver (bilirubin < 1.2 mg/dl), *SNL* survival with native liver, *LF* liver failure/impairment, *RT* radiofrequency ablation, *US* ultrasound sonography, *CEUS* contrast-enhanced ultrasound sonography, *CT* computed tomography, *MRI* magnetic resonance imaging

The median age at Kasai procedure was 66.0 days (IQR 21.8). Following Kasai-procedure information on sequelae of portal hypertension was available for 9 patients (21.4%), no information was available on cholangitis incidence and frequency.

The median age at the time of HCC diagnosis was 4.0 years (IQR 8.8), with the youngest being 7 months and the oldest 34 years. Twenty-two patients (52.4%) were diagnosed during routine follow-up or transplant evaluation, while in 15 cases (35.7%) HCC was incidentally found in the hepatectomy specimen after liver transplantation. In two patients (4.8%) HCC was detected in the postmortem autopsy.

### Liver function tests

Liver function was reported in 22 patients (52.4%) at the time of HCC diagnosis. Of these, 17 (77.3%; 17/22) suffered from progressive jaundice and liver failure, while five patients did not present with jaundice. In the remaining 20 patients (47.6%), no information on liver function was available.

### Tumor markers

Laboratory tests revealed an increased [positive] alpha-fetoprotein (AFP) in 15 patients (15/22; 68.2%) and normal [negative] values in 7 (7/22; 31.8%). For one patient a negative CEA was reported.

### Imaging

Ultrasound findings were reported in 14 patients with suspected malignant lesions in 11 (11/14; 78.6%) cases. In addition, a contrast-enhanced ultrasound (CEUS) was performed in one patient and was highly suspicious for HCC. CT was performed in 14 cases revealing a malignant tumor in 12 patients (12/14; 85.7%). MRI was performed in 7 patients with evidence of a malignant tumor in 6 (6/7; 85.7%) patients.

### Histopathology

For 24 patients (57.1%) histopathology reports were presented (Table 1). In one case, a rare syncytial giant cell variant of HCC in a 7 month old BA patient was reported. Twelve patients (50.0%) had a well-differentiated HCC in the specimens, while in three cases (12.5%) well- to moderately differentiated tumors were diagnosed. Six patients (25.0%) had a moderately-differentiated HCC, while in two (8.3%) a poorly-differentiated HCC was present.

### Outcome

One patient was listed for transplant at the time of the report, 7 were deceased, of whom 4 were from reports before 1972, 28 were alive following liver transplantation without recurrence within a follow-up period of 2 months to 5.7 years.

One patient underwent radiotherapy for HCC without transplantation and was alive recurrence-free at 15 months follow up. The only information on chemotherapy was by Tatekawa et al.[12], reporting on neoadjuvant pirarubicin treatment in one HCC patient and adjuvant therapy with doxorubicin plus uracil-futraful and uracil-futraful monotreatment in two patients.

### Cholangiocarcinoma (CCA)

#### Patient information

Eight case reports on CCA (12.5%) in BA native liver survivors were included (Table 2). Four patients (57.1%) underwent KPE in infancy, in two (28.6%) a choledocho-duodenostomy (CD) was performed for correctable biliary atresia (Ohi type I) of whom one had redo surgery at the age of 54 years converting to a hepatico-jejunostomy, and in one child no BA procedure was performed. Per definition five (62.5%) were intrahepatic CCA (iCCA), of which one was diagnosed with a concomitant HCC in a 39 year old long-term survivor with native liver after Kasai procedure. Another three patients (37.5%) were diagnosed with a perihilar CCA (pCCA). The pCCA diagnosis was made in extrahepatic bile ducts in the patients with CD for Ohi type I BA, while in one patient diagnosis was made at the porta region (anastomotic site).

**Table 2:** Summary of 8 cholangiocarcinoma (CCA) cases in biliary atresia patients

Author	N =	Procedure	Timing of diagnosis	CCA type	Imaging	Laboratory	Age	Liver tests	Outcome
Ohshina et al. 2022 [70]	1	CD	Pre-LTx	pCCA	CT: neg EUS: neg PET/CT: neg Endoscopic cytology + biopsy: pos	n.a	52 yr	SNL	n.a
Uno et al. 2020 [71]	1	KPE	Incidental at LTx	pCCA	CT: negative US: negative	n.a	17 yr	LF	n.r. 15 yr post-LTx
Nio et al. 2019 [72]	1	CD (I) neonatal period HJ(II) at 54 yr	Pre-Tx	pCCA	CT: pos	CEA: positive CA19-9: pos	63 yr	SNL	Deceased
Yoon et al. 2014 [56]	1	KPE	Pre-LTx	iCCA	CT: pos	AFP: neg	13 yr	n.a	Deceased with recurrence 7 m post-LTx (CCA)
Arai et al. 2016 [73]	1	KPE	Pre-LTx	iCCA + HCC	CT: pos	CA19-9: pos	39 yr	LF	Deceased 8 m after diagnosis
Fukuda et al. 2012 [74]	1	KPE	Incidental at LTx	iCCA	CT: neg US: neg	CEA: pos CA19-9: pos	30 yr	LF	Deceased LTx with early recurrence 11mo post-LTx
Vera et al. 2012 [75]	1	KPE	Incidental at LTx	iCCA	US: neg CT: neg	n.a	16 yr	LF	Resection of thoracic metastasis 20 m post-LTx Alive 33 m post-LTx with extensive bone metastases
Kulkarni et al. 1977 [76]	1	None	Explorative Laparotomy	iCCA	n.a	n.a	11 yr	LF	Deceased

*LTx* liver transplantation, *SNL* survival with native liver, *LF* liver failure/impairment, *pCCA* perihilar CCA, *iCCA* intrahepatic iCCA, US ultrasound sonography, *CEUS* contrast-enhanced ultrasound sonography, *CT* computed tomography, *PET* positron emission tomography, *EUS* endoscopic ultrasound

The median age at the time of CCA diagnosis was 23.5 years (IQR 27.0), with the youngest being 11 years and the oldest 63 years. Four patients (50.0%) were diagnosed before LTx, while in three (37.5%) the hepatectomy specimen during LTx confirmed CCA. In one child (12.5%) reported by Kulkarni et al. in 1977 no surgical procedure was performed for BA, however, the child underwent explorative laparotomy at the age of 11 years in the process of liver failure revealing the CCA.

### Liver function tests

Five of the 8 patients presented with progressive jaundice and liver failure at the time of CCA diagnosis, while 2 showed a stable liver function. For one patient no information on liver function at diagnosis was available.

### Tumor markers

In cases in which CA 19–9 was available (n = 3) it was always pathologically increased.

### Imaging

Ultrasound was negative for malignant findings in all 3 reported cases. CT was negative in 3 patients (3/7; 42.9%) and positive in 4 (4/7; 57.1%). In one patient a negative PET/CT and in another a negative EUS was performed. In one patient with CD repair for BA endoscopic cytology and mapping biopsies were performed, confirming the CCA.

### Outcome

One patient was recurrence-free 15 months following LTx and one was alive at the time of the report 33 months post-LTx with extensive bone metastasis. Two patients deceased early within the first year post-LTx, of whom one received a cisplatine plus gemcitabine chemotherapy for recurrence, while two patients died pre-LTx receiving a palliative chemotherapy, of whom one had a diagnosis of a concomitant HCC. One patient died post-LTx without further details and for one patient no outcome data was available.

### Hepatoblastoma (HB)

#### Patient information

Five HB cases (9.1%) were identified in the literature review. All patients underwent KPE during early infancy. Three HB (60.0%) were diagnosed during routine follow-up, while two (40.0%) were incidental findings at LTx in the hepatectomy specimen.

### Liver function tests

Information on the liver function prior to HB diagnosis is scarce, but one patient was reported to be in a stable native liver situation, but later deceased due to tumor rupture under neoadjuvant chemotherapy with SUPER-PLADO [13]. The other reports included information on liver failure during follow-up.

### Tumor markers

AFP was positive in all reports with available information.

### Imaging

Imaging was positive in 4, including ultrasound and CT (of which one was diagnosed in retrospect after positive HB diagnosis in hepatectomy specimen). In one patient no information on imaging was available.

### Outcome

Post-transplant information with recurrence-free intervals from one month to 6 years (n = 3) and no post-transplant recurrences (n = 3) were reported. The only report on adjuvant post-transplant chemotherapy is from Tatekawa et al. in 2001, mentioning treatment with pirarubicin and paraplatin [12].

### Miscellaneous

Recently, the incidental finding of a small intestinal adenocarcinoma (ADC) at the anastomotic site of the hepato-jejunostomy in a 49 year old was reported (Table 3) [14]. After a long interval of post-KPE native liver survival, sudden onset of jaundice was reported. In the post-LTx specimen the ADC was detected. Preoperative imaging, including MRI and CT studies were negative, however, positive CEA was available. The patient was recurrence-free at 10 months post-LTx follow-up.

**Table 3:** Information on different cancer types, including hepatoblastoma (HB), intestinal adenocarcinoma (ADC) and papillary cancer of the thyroid gland (PTC) in biliary atresia patients following Kasai-procedure

Author	N =	Procedure	Carcinoma	Finding	Diagnostics	Age	Liver-Function	Outcome	(Neo) Adjuvant chemo
Ishikawa et al. 2023 [14]	1	KPE	Intestinal ADC	Incidental at LTx	CT: neg MRI: neg CEA: pos	49 yr	LF	n.r. 10mo post-LTx	No chemo
Amir et al. 2016 [77]	2	KPE	HB	Incidental at LTx	US: neg CT: pos AFP: pos	38mo (I) 17mo(II)	LF	n.r.: 6 yr (I) & 20mo (II) post-LTx	No chemo
Taat et al. 2004 [13]	1	KPE	HB	n.i	CT: pos AFP: pos	2 yr	SNL	Deceased Tumor rupture	Neoadjuvant chemo SUPERPLADO (CDDP, carboplatin, doxorubicin)
Tatekawa et al. 2001 [12]	1	KPE	HB	Pre-Tx	CT: pos AFP: pos	4 yr	n.a,	n.r. 1mo	Pirarubicin + paraplatin
Sato et al. 2000[62]	1	KPE	HB	Pre-Tx	US: pos	4 yr	n.a	n.a	n.a
Marquardt et al. 1979 [15]	1	KPE	PTC thyroid gland	No surgery Post mortem autopsy	n.a	5 yr	LF	Deceased	n.a

*LTx* liver transplantation, *SNL* survival with native liver, *LF* liver failure/impairment, *RT* radiofrequency ablation, *US* ultrasound sonography, *CT* computed tomography, *MRI* magnetic resonance imaging

Marquardt et al. reported on a case of papillary thyroid cancer in a BA patient in 1979, diagnosed in post-mortem autopsy [15]. The patient did not undergo surgery, neither KPE nor LTx and deceased at the age of 5 years.

## Discussion

Increasing reports on malignancies in BA patients, from early childhood to late adulthood, emphasize the necessity of surveillance strategies. We identified 55 cases of malignancies in native liver survivors, mainly referring to hepatocellular carcinoma (HCC), cholangiocarcinoma (CCA) and hepatoblastoma (HB) diagnosis in the descending order.

While CA19-9 for CCA and AFP for HB have been pathologically elevated in all cases, with the limitation of missing data in some reports, HCC diagnosis has proven to be difficult, with AFP being positive in around 68% of scenarios and imaging techniques including CT, MRI, US and CEUS presenting with limitations in their sensitivity. The differentiation of malignancies from macroregenerative nodules in liver fibrosis and cirrhosis is particularly challenging even for specialized radiologists.

### HCC surveillance strategies

Reports on HCC in BA within the first year of life are emerging and standardized monitoring protocols in pediatric chronic liver diseases are missing. The median age at diagnosis in our review was 4.0 years, leading to the conclusion that early surveillance is necessary. In adults with chronic liver diseases, especially hepatitis B and C patients, surveillance strategies are a crucial part of monitoring, resulting in slightly differing protocols from the AASLD, EASL, APASL and JSH [16–20]. Variations in the protocols are based on age profiles, racial background, manifest cirrhosis and hepatitis serology, however, for the target populations ultrasound is recommended every 6 months [16]. The APASL adds determination of AFP in 6 months intervals, while the JSH includes Lens culinaris agglutinin A-reactive fraction of AFP (AFP-L3%) and serum des-gamma-carboxy-prothrombin (DCP) in their 6 months monitoring [19, 20].

AFP-L3% is derived from malignant transformed hepatocytes and a diagnostic sensitivity from 75.0% to 96.0% for HCC has been reported [21, 22]. However, in a recent meta-analysis by Zhou et al. the authors concluded a high specificity and low sensitivity for the diagnosis of early HCC, therefore questioning its plausibility in surveillance strategies [23]. Reports on AFP-L3% in pediatric hepatic tumors are scarce and limited to reports on the monitoring for HB recurrence after resection [24].

DCP is associated with an acquired defect of the post-translational carboxylation of prothrombin precursors in malignant cells and a sensitivity from 30 to 90% for HCC diagnosis has been reported [25]. A large-scale, Chinese multicenter study demonstrated a higher accuracy for HCC detection with DCP compared to AFP in hepatitis B patients [26]. Schreuder et al. recently reported on their experience with DCP in the HCC diagnosis of two adolescents with glycogen storage disease type Ia (GSDIa), in whom AFP remained normal [27]. The authors recommended the DCP screening for HCC surveillance in GSDIa patients.

A combination of these strategies has been introduced with GALAD, including gender, age, AFP-L3% and DCP [28, 29]. Despite ongoing evaluations in adult cohorts, a sensitivity of only 65% and specificity of 85% has been achieved. Berhane et al. demonstrated a sensitivity of 60 – 80% for early HCC in a multinational case control study including 2.430 HCC and 4.404 patients with chronic liver diseases, making it a promising model for HCC surveillance [30]. However, El-Serag et al. recently published the modification of the hepatocellular carcinoma early detection screening (HES) score [31]. The HES score combines AFP, age, alanine aminotransferase and platelets, and has been superior for early HCC detection compared to AFP alone. The novel HES V2.0 further includes AFP-L3 and DCP into the score and has shown to outperform individual markers and the GALAD in a phase 3 biomarker study for early HCC detection.

Multi-analytic designs, combining serum markers and cfDNA methylation state and alterations, offer further tools for HCC diagnosis in adults through multi-target blood tests [32]. The Oncoguard Liver platform reached an overall sensitivity of 80% and early stage-sensitivity of 72% for HCC diagnosis through a screening for methylation markers (HOXA1, TSPYLS, EMX1, B3GALT6), AFP and AFP-L3% [33].

A novel and promising approach are liquid biopsies, detecting circulating tumor cells, DNA and extracellular vesicles for cancer diagnosis [32, 34]. Liquid biopsies based on cfDNA-targeted exome sequencing might not only allow for early detection, but could even help with treatment stratification. Ikeda et al. reported on 14 patients with advanced HCC, in whom liquid biopsies were performed and identified somatic alterations in all [35]. Twelve out of 14 patients had at least one actionable genomic alteration and two patients received treatment based on the ctDNA results showing positive effects, in one patient with detectable DCP decline and in one with AFP decline and stable diseases with central necrosis of the tumor in the imaging. Most important, the authors identified a heterogenous genomic profiling in all HCC patients, emphasizing the necessity of individualized treatment strategies. However, with the limited data on solid tumor surveillance, especially in children, liquid biopsies currently do not seem to go beyond a hopeful outlook.

### Surveillance strategies for other malignant entities

Cholangiocarcinoma (CCA) are rare tumors with an overall incidence of less than 2/ 100,000, but they are the second most common primary liver tumor following HCC [36].

CCA is known as an aggressive tumor, mostly diagnosed in an advanced stage and is therefore associated with a poor prognosis [37]. In the current literature, median overall survival of 5% months is reported for pCCA and approximately 7% for iCCA [38, 39].

While several risk factors have been identified, e.g. parasitic infections and primary sclerosing cholangitis in adults, choledochal cysts and malformations have been the most important in pediatric cohorts [36, 40, 41]. Although the association of CCA and chronic biliary inflammation is well-known, BA has not yet been identified as a potential origin for CCA. Numerous surveillance strategies for CCA in specific cohorts in adults have been suggested and recently the cholangiocarcinoma screening and care program (CASCAP) has been introduced [42]. Part of the screening program are ultrasound examinations every 6 months and a low threshold for sectional imaging in case of uncertainties.

In the presented BA cohort with CCA, CA 19–9 was available in only three, however, all had increased levels. CA19-9 as a tumor marker for CCA has been reported to achieve sensitivities and specificities of 100% and 94% in some studies, and it has been discussed that CA19-9 increases the positive and negative predictive value of other screening methods [41].

In case of Hepatoblastomas (HB), alpha-fetoprotein (AFP) is an established marker for diagnosis, being positive in about 90% of patients [43]. In the detected BA patients with HB diagnosis, AFP was positive in all available reports. In addition, AFP has not only been used for diagnosis, but has recently been advocated as a surveillance marker for relapse in children with AFP-positive hepatoblastomas [44]. For certain risk groups for early HB diagnosis (e.g. Beckwith-Wiedemann syndrome or Simpson-Golabi Behmel) abdominal ultrasounds and AFP screenings every 3 months have been recommended, starting from birth [45].

### Suggested follow-up protocol

Despite novel developments in HCC and HB surveillance and monitoring, ultrasound and AFP controls every 3–6 months following Kasai procedure remain the most effective instruments for surveillance, especially learning from strategies in adult cirrhotic patients by global hepatological associations. Based on the reports on HCC diagnosis in BA infants within the first year of life[46–48], monitoring should already begin in the aftermath of Kasai procedure, especially in infants with early Kasai failure not clearing their jaundice postoperatively.

While data in children is scarce, AFP-L3% and DCP might already be included for HCC surveillance during childhood. With transition into adult care, novel models like GALAD or HES v2.0 represent additional possible surveillance strategies based on experiences in adults with liver cirrhosis.

In addition, annual CA19-9 monitoring may be added in early adolescence for CCA monitoring in BA native liver survivors, as the youngest cases in the literature were 11 and 13 years at diagnosis.

## Data Availability

No datasets were generated or analysed during the current study.
